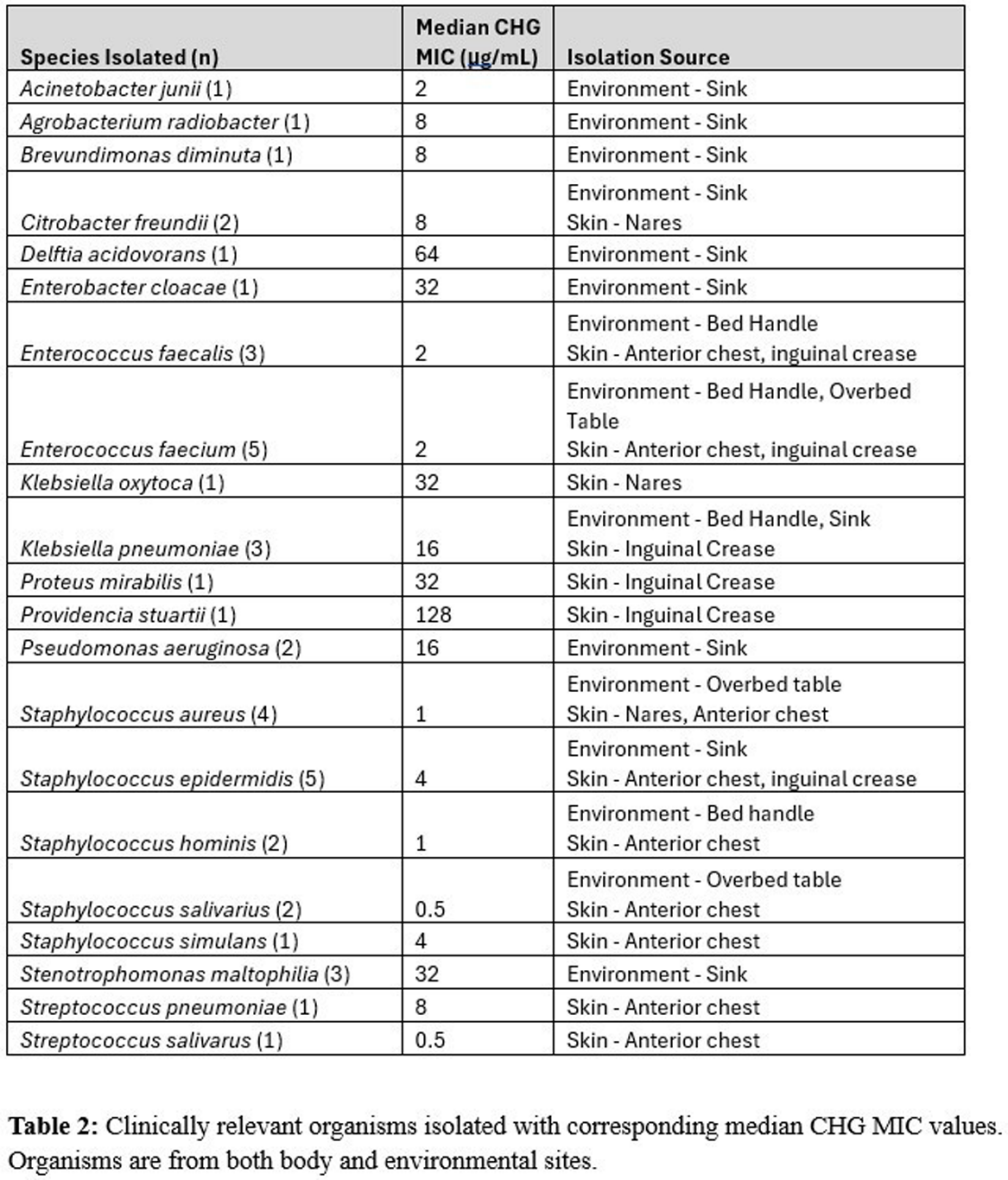# 305 Adherence to an Electronic Medical Record Alert for Repeat Blood Cultures within 48 Hours

**DOI:** 10.1017/ash.2026.10659

**Published:** 2026-06-23

**Authors:** Rachel Medernach, Ellen Gough, Mckenzi King, Mary Carl Froilan, Mackenzie May, Lahari Thotapalli, Michael Schoeny, Sarah Sansom, Erica Hartmann, Mary Hayden

**Affiliations:** 1 Rush University Medical Center; 2 Rush University; 3 Northwestern University

## Abstract

**Background** Bacterial resistance to frequently used antimicrobial therapies is a significant threat to human health. Chlorhexidine digluconate (CHG) is a topical antiseptic used to reduce healthcare-associated infections, including those caused by multidrug-resistant organisms. There is concern that exposure to CHG may lead to elevated bacterial CHG minimum inhibitory concentrations (MICs) in healthcare environments where the antiseptic is used. We investigated whether skin bacteria of CHG-exposed patients had elevated CHG MICs compared to patients without CHG exposure. Additionally, we evaluated whether bacteria isolated from environmental surfaces of CHG-exposed patients and from surfaces with detectable concentrations of CHG had higher CHG MICs compared to bacteria isolated from room surfaces without CHG exposure or from surfaces without detectable CHG. Methods We recruited a prospective observational cohort including two patient groups: 15 patients without CHG exposure during hospitalization, and 15 patients with daily CHG bathing for ?14 days prior to enrollment. Informed consent was obtained, and clinical data were recorded. Skin and environmental swab samples were collected using E-swabs and sponge sticks respectively and cultivated on non-selective media (Figure 1). A subset of isolates underwent CHG and antibiotic MIC determination by microbroth dilution. Swab samples of the same locations were collected using pre-moistened sterile water swabs and processed via colorimetric assay. Data were analyzed using multilevel models. Results No significant clinical differences between groups were noted, including in antimicrobial exposure or infection history. 360 unique bacterial isolates were identified, with Staphylococcus epidermidis and Staphylococcus hominis being the most common organisms isolated from all skin and environmental sites (Table 1). CHG MICs were not significantly different across all organisms detected at all sites in exposed versus non-exposed groups (p=0.4) (Table 2). No residual CHG was found on skin or environmental surfaces of the CHG non-exposed group. Residual CHG in the exposed group was most frequently detected on the anterior chest (n=13/15) and bed handle (n=8/15), and levels of residual CHG on body sites were significantly elevated compared to environmental sites (p=<0.0001). Bacteria found where residual CHG was detected on both skin and environmental sites did not have significantly elevated CHG MICs (p=0.21). Conclusion We found no evidence that CHG exposure correlates with elevated CHG MICs of skin or environmental bacteria, suggesting routine CHG use to prevent infections does not contribute to resistance. This study is the first to correlate bacterial CHG MICs with residual environmental CHG, and to perform colorimetric CHG testing on environmental surfaces.

**Figure:**
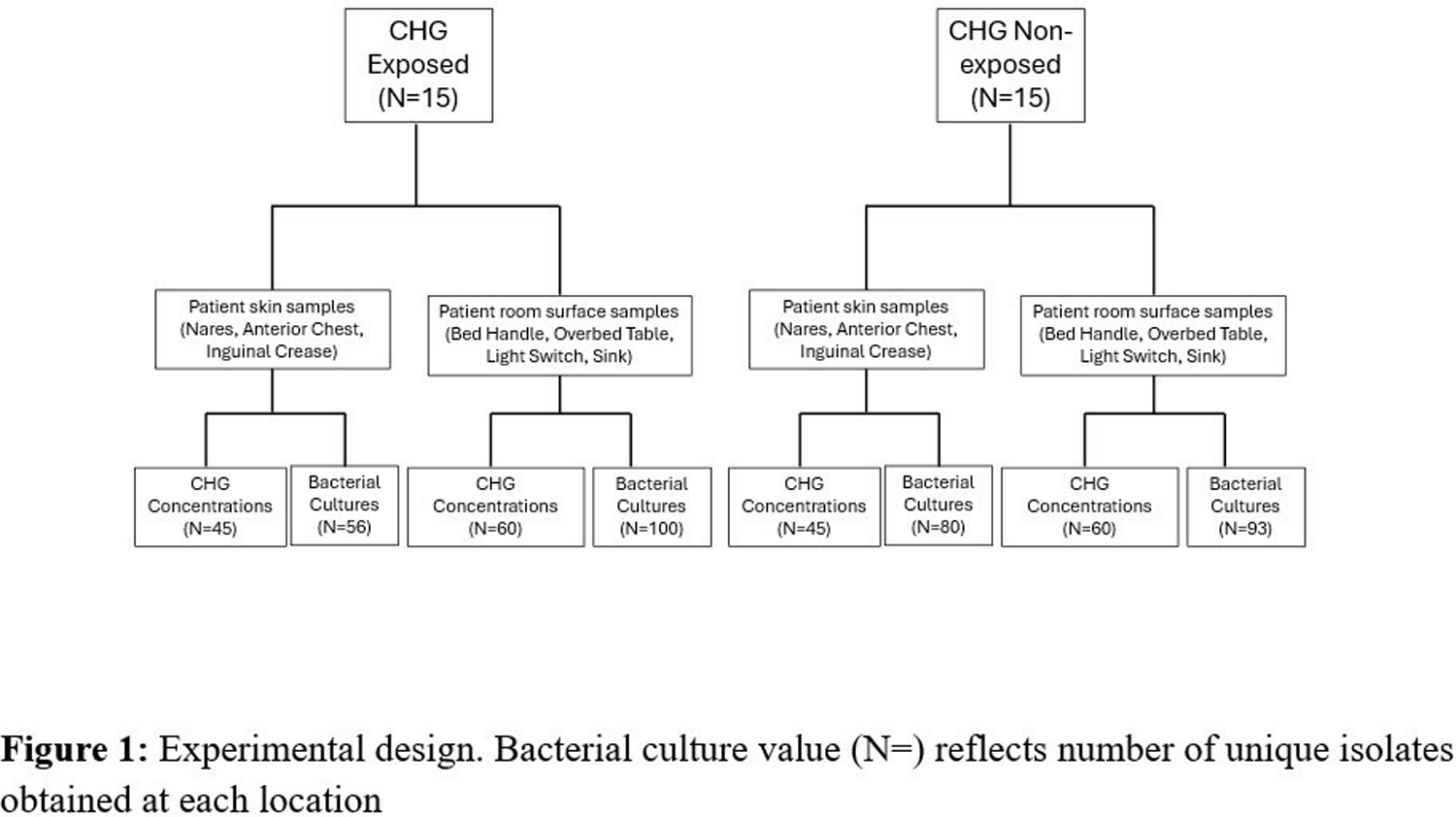

**Figure:**
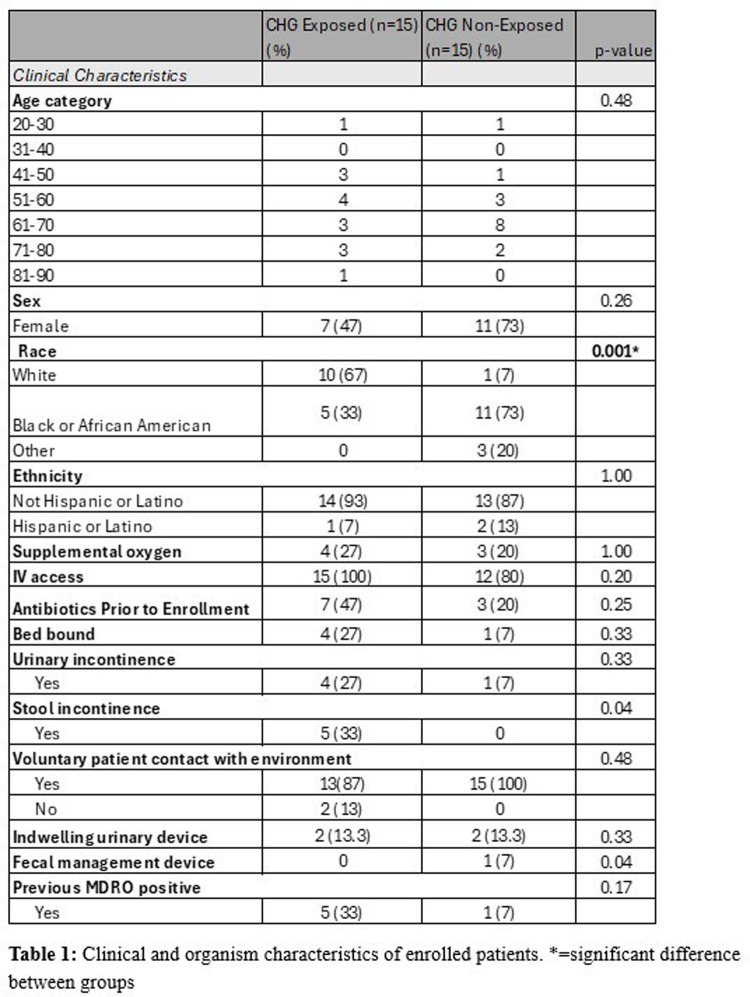

**Figure:**